# Miscellany

**Published:** 1902-10

**Authors:** 


### MISCELLANY.

THE Maltine Company announces that 280 essays on "Preventive Medicine" have been entered in competition for the two cash prizes — $1,000 and $500, respectively — which that firm offered last February. These essays are now in the hands of the three judges, Dr. Daniel Lewis, of New York, Dr. Chas. A. L. Reed, of Cincinnati, and Dr. John Edwin Rhodes, of Chicago, and their decision is awaited with great interest by the medical profession.

---

FOUR HUNDRED DOLLAR PRIZE.—Dr. J. B. Mattison, Medical Director, Brooklyn Home for Narcotic Inebriates, offers a prize of $400 for the best paper on the subject: Does the habitual subdermic use of morphia cause organic disease? If so, what?

Award to be determined by a committee: T. D. Crothers, Hartford, editor *Journal of Inebriety*, chairman; J. M. Van Cott, professor of pathology, Long Island College Hospital, Brooklyn, and Dr. Wharton Sinkler, neurologist to the State Asylum for the chronic insane, Philadelphia.

All papers must be in the hands of the chairman on or before December 1, 1903, to become the property of the American Association for the Study and Cure of Inebriety, and to be published in such journals as the committee may select.

---

FRACTURE of the humerus above the condyles, is the subject of Chart No. 9, issued by Battle & Co., of St. Louis. The bones and muscles that are involved or play a part in causing the deformity resultant from this fracture are well depicted in color, making a useful guide to the student in studying the injury. Charts will be furnished on application to the publishers.

---

### Late News Item.

THE President appears to have sustained a more serious injury in the Pittsfield accident than was supposed at first, or than he admitted to himself. It is to be regretted that it rendered an abandonment of his itinerary a necessity, but it is fortunate that surgical intervention was so seasonably invoked at Indianapolis. The serous accumulation was released so promptly and the President being enjoined absolute rest, gives promise of his speedy recovery. Even the reopening of the wound that is announced at this writing, and the surgical treatment of the periosteum may not mean more than a little further delay in so robust a person —at all events, let us so confidently hope and believe.

